# Randomized controlled trial of physical activity intervention effects on fatigue and depression in multiple sclerosis: Secondary analysis of data from persons with elevated symptom status

**DOI:** 10.1016/j.conctc.2020.100521

**Published:** 2020-01-14

**Authors:** Robert W. Motl, Brian M. Sandroff

**Affiliations:** Department of Physical Therapy, School of Health Profession, University of Alabama at Birmingham, Birmingham, AL, USA

**Keywords:** Physical activity, Multiple sclerosis, Depression, Fatigue

## Abstract

**Background:**

Physical activity interventions have yielded reductions in fatigue and depressive symptoms among persons with multiple sclerosis(MS) who have not been screened for elevated baseline symptoms scores.

**Purpose:**

This short communication describes a secondary analysis of data from a previous randomized controlled trial (RCT) and focused on physical activity intervention effects on fatigue and depression among persons with MS who had elevated baseline symptom scores.

**Method:**

Of the 76 persons who completed the RCT, 64(84%) had baseline fatigue severity scale(FSS) scores indicating elevated levels of fatigue(n = 30,intervention; n = 34,control), and 26(34%) had baseline hospital anxiety and depression scale, depression(HADS-D) scores indicating elevated depressive symptoms (n = 13,intervention; n = 13,control). The physical activity intervention was delivered over a 6-month period, and the control condition was a 6-month waitlist. The participants completed the FSS and HADS-D as part of baseline and follow-up battery of assessments.

**Results:**

There was a statistically significant change in FSS scores favoring the physical activity intervention, and the effect size of 0.73 was larger than reported in a previous meta-analysis of RCTs of physical activity and fatigue in MS of 0.45(95%CI=.22,.68). We observed a statistically significant change in HADS-D scores favoring the physical activity intervention, and the effect size of 1.21 was larger than reported in a previous meta-analysis of RCTs of physical activity and depression in MS of 0.36(95%CI=.18,.54).

**Conclusion:**

Such results provide preliminarily support for the application of physical activity interventions for the “treatment” of fatigue and/or depression in MS, pending subsequent confirmatory efficacy or effectiveness trials.

## 1 Introduction

There is consistent evidence from meta-analyses of randomized controlled trials(RCTs) that physical activity interventions have yielded reductions in fatigue and depression among persons with multiple sclerosis(MS) [1,2]. One notable limitation of the RCTs in those meta-analyses is the overall lack of focal inclusion of persons with MS who have elevated fatigue or depressive symptoms [3]; this limits our understanding of physical activity as a treatment for fatigue and depression in MS. For example, we previously reported that an Internet-delivered physical activity intervention yielded statistically significant reductions in fatigue and depressive symptoms in a RCT of 82 persons with MS [4]. Importantly, the participants in that study were prescreened, in part, based on physical activity levels, but not fatigue or depressive symptoms.

This short communication describes a secondary analysis of data from the single**,** aforementioned RCT [4] and examined the possibility that the effect of the physical activity intervention on fatigue and depression would be larger among participants purposefully selected based on elevated baseline symptom scores; this is necessary for testing the hypothesis that participants in that previous RCT who had elevated baseline levels of fatigue and depression would report larger effects of the physical activity intervention on those respective symptoms than documented in previous meta-analyses [1,2] or the original report from the aforementioned RCT [4]. Accordingly, we selected subsamples of participants with elevated baseline levels of fatigue and depression, and then examined the effect of the physical activity intervention compared with the waitlist control condition on those symptoms among the participants with elevated baseline symptomology.

## 2 Materials and methods

### 2.1 Participants

This short communication reports on a secondary analysis of data from a previous RCT [4], and further information on the study design, procedures, and participants is provided in the original paper. The inclusion criteria included age of 18–64 years, diagnosis of MS, relapse-free for the past 30 days, Internet access, ambulatory, minimal risk for engaging in physical activity along with physician approval, and physical inactivity. This paper included a secondary analysis of data from subsamples selected based on cut-points for elevated baseline levels of fatigue and depression.

### 2.2 Outcomes of interest

#### 2.2.1 Fatigue

The Fatigue Severity Scale (FSS) [5] was the measure of fatigue. The FSS includes 9 items that measure patient-perceived fatigue over the past week. The items are rated on a scale between 1 and 7. The item scores are averaged into a summary score, and we applied the FSS scale cut-point of 4 for generating the subsample with elevated baseline levels of fatigue [5]. Importantly, we focused on the FSS as a measure of fatigue in this secondary analysis for two primary reasons. The first is that the FSS is the most common measure of fatigue in RCTs of physical activity in MS [2]. The second is that the FSS has captured clinically meaningful changes in fatigue in previous RCTs of physical activity in MS [2].

#### 2.2.2 Depression

The Hospital Anxiety and Depression Scale(HADS) [6] was the measure of depressive symptoms. This scale has 14 items that measure the frequency of anxiety and depression symptoms over the past 4 weeks; we only included the depression subscale(HADS-D). The items are rated on a scale between 0 and 3, and there are two reverse-scored, negatively-phrased items. The item scores are then summed into a summary score, and we applied the HADS-D subscale cut-point of 8 for generating the subsample with elevated baseline levels of depressive symptoms [6].

#### 2.2.3 Participant characteristics

Participants provided information on age, sex, and disease type and duration. We measured height and weight using a scale stadiometer. We administered the Patient Determined Disease Steps(PDDS) [7] scale for characterizing disability status.

### 2.3 Physical activity intervention

The physical activity intervention was delivered over a 6-month period with the goal of increasing lifestyle physical activity, primarily walking, among a sample of persons with MS [4]. This intervention had several components including a study website with information about becoming more physically active based on social cognitive theory, self-monitoring and goal setting using a pedometer and activity logs, and one-on-one web-based video coaching sessions with a behavioral coach. The control condition was a waitlist control, and all participants in this condition received the physical activity intervention upon completion of the study.

### 2.4 Procedures

All study procedures were approved by a university institutional review board. Interested participants contacted the research team and were screened for eligibility. The screening process involved assessment of inclusion criteria followed by medical clearance from a physician and signed informed consent. Participants then provided demographic and clinical information and completed a baseline battery of assessments including the FSS and HADS-D in the laboratory. The project coordinator randomized participants into the intervention or control condition and provided instructions regarding the assignment via mail and email. Participants completed the assigned condition over the 6-month period of the study and then the follow-up battery of assessments including the FSS and HADS-D.

### 2.5 Data analysis

Analysis were performed using SPSS statistics 24(IBM, Inc., Armonk, NY). Descriptive statistics are provided as mean(SD), unless otherwise noted. The analysis involved separate condition(physical activity intervention and waitlist control) by time(baseline and follow-up) mixed-factor ANOVAs on FSS and HADS-D scores. The mixed-factor ANOVAs were appropriate as the model contained a single, between-subjects factor with two levels (i.e., physical activity intervention and waitlist control conditions) and a single, within-subjects factor with two levels or repeated measurements (i.e., baseline and follow-up measurements). We expressed effect sizes for pre-post changes in FSS and HADS-D scores between intervention and control conditions as Cohen's *d* for comparison with previous meta-analyses [1,2] and the original, published RCT, and the effect sizes were computed such that a positive effect indicated a larger change in the intervention than control condition. The *d* values were interpreted as small, moderate, and large based on guidelines of 0.2, 0.5, and 0.8, respectively [8].

## 3 Results

### 3.1 Participants

There were 82 participants who were randomly assigned into the conditions, and 37 completed the physical activity intervention and 39 completed the waitlist control; there were 4 drop-outs from the intervention condition and 2 drop-outs from the control condition. Of the 76 persons who completed the study, 64(84%) had baseline FSS scores indicating elevated levels of fatigue, and 26(34%) had baseline HADS-D scores indicating elevated depressive symptoms, respectively. Those percentages approximate the proportion of MS patients with elevated FSS and HADS-D scores [5,9]. The descriptive and clinical characteristics of the subsamples within the physical activity intervention and waitlist control conditions are provided in Table 1.

**Table 1:** Participant characteristics and tests for baseline differences between physical activity intervention and control groups per subsample with elevated fatigue or depression symptom scores. Values are means (standard deviation) unless otherwise noted.

Variable	Fatigue Subsample (n = 64)			Depression Subsample (n = 26)		
	Intervention (n = 30)	Control (n = 34)	*p* value	Intervention (n = 13)	Control (n = 13)	*p* value
Age, years	48.7 (9.1)	50.1 (8.8)	.54	50.5 (10.2)	50.3 (7.8)	.97
Sex, female/male	22/8	26/8	.77	9/4	7/6	.42
Height, cm	168.8 (9.3)	168.1 (7.0)	.75	167.9 (8.5)	172.7 (6.7)	.12
Weight, kg	78.7 (17.0)	75.3 (15.3)	.40	81.6 (18.6)	79.0 (14.6)	.69
MS Type, n/% RRMS	21/70%	27/79%	.39	9/69%	9/69%	1.00
PDDS, mdn (IQR)	3.0 (3.0)	3.0 (2.0)	.71	3.0 (2.5)	3.0 (2.0)	.80
Disease duration, years	10.4 (7.1)	13.6 (9.1)	.12	10.0 (7.2)	12.9 (8.4)	.36
Symptoms Scores						
FSS	5.6 (0.8)	5.6 (0.9)	.99	n/a	n/a	n/a
HADS-D	n/a	n/a	n/a	10.6 (3.0)	10.9 (2.8)	.79

Note: FSS, Fatigue Severity Scale; HADS-D, Hospital Anxiety and Depression Scale, Depression Subscale; RRMS, relapsing-remitting multiple sclerosis; PDDS, Patient-Determined Disease Steps; SR-EDSS, Self-reported Expanded Disability Status Scale.

### 3.2 Fatigue

**Fig. 1:**
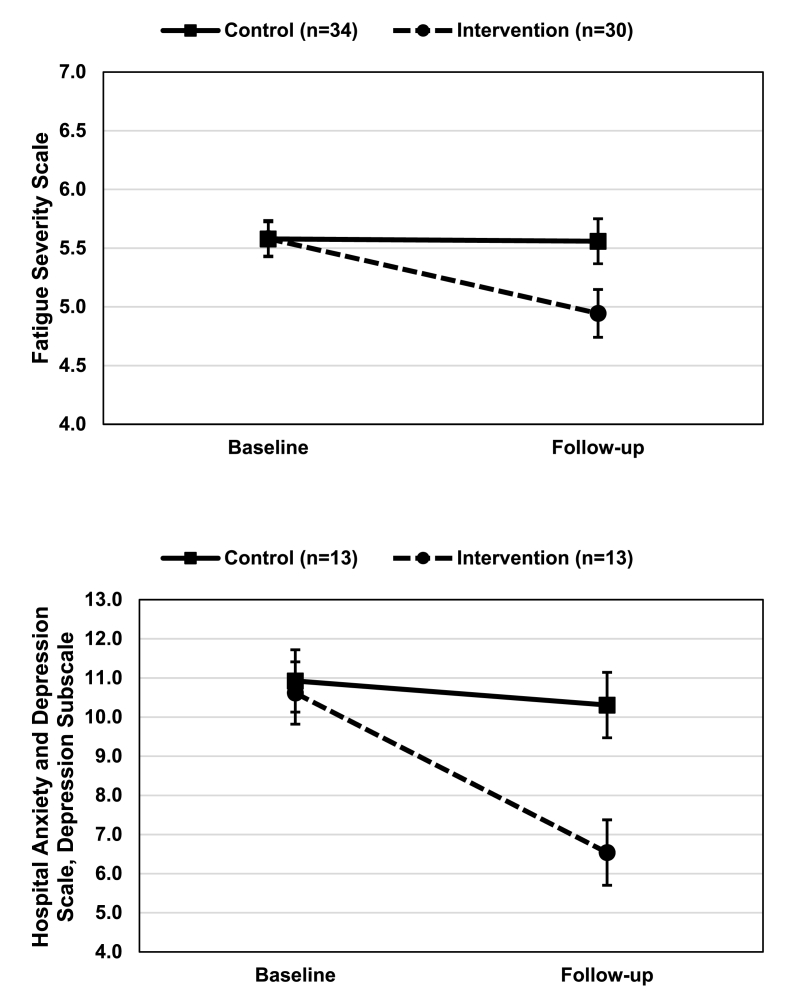
Effects of the physical activity intervention on fatigue (Top panel) and depressive symptoms (Bottom panel) compared with control in persons with MS who had elevated baseline symptom scores. Values are means with standard errors.

There was a statistically significant condition by time interaction on FSS scores in the subsample with elevated baseline levels of fatigue(*F*_1,62_ = 9.57,*p* = .003); see Fig. 1. There was a moderate-to-large reduction in FSS scores favoring the physical activity intervention compared with the waitlist control(*d* = 0.73). The mean (SD) FSS scores for those in the intervention condition for pre-test and post-test were 5.6(0.8) and 4.9(1.2), respectively, whereas the mean (SD) FSS scores for those in the waitlist control condition for pre-test and post-test were 5.6(0.9) and 5.6(1.1), respectively.

### 3.3 Depression

There was a statistically significant condition by time interaction on HADS-D scores in the subsample with elevated baseline levels of depressive symptoms(*F*_1,24_ = 6.54,*p* = .017); see Fig. 1. There was a large reduction in HADS-D scores favoring the physical activity intervention compared with the waitlist control(*d* = 1.21). The mean (SD) HADS-D scores for those in the intervention condition for pre-test and post-test were 10.6(3.0) and 6.5(2.9), respectively, whereas the mean (SD) FSS scores for those in the waitlist control condition for pre-test and post-test were 10.9(2.8) and 10.3(3.1), respectively.

## 4 Discussion

There is consistent evidence that physical activity interventions yield reductions in fatigue and depressive symptoms among persons with MS [1,2], but there is a lack of inclusion of persons who have significant fatigue or depressive symptoms in those RCTs [3]. This paper involved a secondary analysis of data from a single, previously published RCT [4] and examined the possibility that the effect of the physical activity intervention on fatigue and depression was larger among those with elevated baseline symptom scores. We observed a moderate-to-large reduction in fatigue scores in the subsample of persons with FSS scores that exceeded the cut-point for elevated levels of fatigue, and a large reduction in depression scores in the subsample of persons with HADS-D scores that exceeded the cut-point for elevated levels of depression. Such results provide preliminarily support for the application of physical activity interventions for the “treatment” of fatigue and/or depression in MS, pending subsequent confirmatory efficacy and effectiveness trials.

We compared the effect sizes for reductions in fatigue and depression in this study against effect sizes reported in meta-analyses [1,2]. For example, we observed a moderate-to-large reduction in FSS scores based on an effect size of 0.73, and this was larger than reported in a meta-analysis of RCTs of physical activity and fatigue in MS of 0.45(95% CI=.22,.68) [2]. We further observed a large reduction in HADS-D scores based on an effect size of 1.21, and this was larger than reported in a meta-analysis of RCTs of physical activity and depression in MS of 0.36(95% CI=.18,.54) [1]. One reason for the larger effect sizes in this study might be the selection of subsamples with FSS and HADS-D scores that exceed cut-points indicative of elevated symptomology. Indeed, the effect sizes herein for FSS and HADS-D of 0.73 and 1.21, respectively, were larger in this secondary analysis of only subsample with elevated symptomology than the effect sizes reported in the main outcomes paper [4] that included all participants, regardless of baseline symptomology scores. That paper reported effect sizes for FSS and HADS-D of 0.49 and 0.40, respectively. The subsamples with elevated symptomology might have had more room for improvement as the scores were higher before the physical activity intervention. We do not believe that this was the result of regression toward the mean as there were minimal changes in scores from the control condition. It further is unlikely that the present pattern of results were attributable to baseline differences in participant characteristics.

This study involved a secondary analysis of data from a single RCT, and the sample in the original study was not prescreened for either fatigue or depression. This is essentially an “after the fact” study and research question, yet it provides preliminary data for confirmation in a future, a priori designed RCT. This study grouped subjects based on self-report measures of fatigue, rather than a clinical diagnosis. Future research might prescreen on clinical measures of fatigue and depression, and then examine the effect of physical interventions on a battery of self-report measures of fatigue and depression for a comprehensive assessment of anti-fatigue and anti-depressant effects of physical activity.

Overall, this study provides preliminary, but promising, results regarding larger effects of physical activity interventions on symptoms of fatigue and depression in persons with MS who were selected based on cut-points for elevated symptomology. These data support future, purposefully-designed RCTs of physical activity interventions for treatment of fatigue and depression in MS, as this has not been the focus of physical activity interventions included in meta-analyses of RCTs in this population. There further may be clinical implications of these preliminary results in that healthcare providers might encourage free-living physical activity among patients with MS who have elevated fatigue and/or depressive symptoms as an approach for treatment of those symptoms.
